# Detecting Malware by Analyzing App Permissions on Android Platform: A Systematic Literature Review

**DOI:** 10.3390/s22207928

**Published:** 2022-10-18

**Authors:** Adeel Ehsan, Cagatay Catal, Alok Mishra

**Affiliations:** 1 Department of Computer Science & Engineering, Qatar University, Doha 2713, Qatar; 2 Informatics and Digitalization Group, Faculty of Logistics, Molde University College-Specialized University in Logistics, 6410 Molde, Norway

**Keywords:** malware detection, static analysis, hybrid analysis, permissions analysis

## Abstract

Smartphone adaptation in society has been progressing at a very high speed. Having the ability to run on a vast variety of devices, much of the user base possesses an Android phone. Its popularity and flexibility have played a major role in making it a target of different attacks via malware, causing loss to users, both financially and from a privacy perspective. Different malware and their variants are emerging every day, making it a huge challenge to come up with detection and preventive methodologies and tools. Research has spawned in various directions to yield effective malware detection mechanisms. Since malware can adopt different ways to attack and hide, accurate analysis is the key to detecting them. Like any usual mobile app, malware requires permission to take action and use device resources. There are 235 total permissions that the Android app can request on a device. Malware takes advantage of this to request unnecessary permissions, which would enable those to take malicious actions. Since permissions are critical, it is important and challenging to identify if an app is exploiting permissions and causing damage. The focus of this article is to analyze the identified studies that have been conducted with a focus on permission analysis for malware detection. With this perspective, a systematic literature review (SLR) has been produced. Several papers have been retrieved and selected for detailed analysis. Current challenges and different analyses were presented using the identified articles.

## 1. Introduction

Mobile phones have become a constant, everyday companion of human users. As reported by Statista [1], the number of users currently owning smartphones is six billion, which is expected to grow by hundreds of millions in the few coming years. From making calls and sending messages to operating financial accounts, smartphones are making everyday tasks possible right from the palm of the hands. This capability has come with great challenges. Users are accustomed to storing sensitive and non-sensitive data on smartphones, since they carry them all the time. This has created a false sense of safety: believing that everything is safe because the smartphone always stays with the owner. Little do users know, smartphones are exposed to all kinds of attacks these days. Currently, there is a great number of apps available for users to install and benefit from. Several of the available apps are malicious, aiming to detect and steal the user’s information from the smartphone without the user’s knowledge. Users unknowingly install these apps, which qualify as malware and have malicious objectives targeting sensitive data. iOS and Android are the two mobile operating systems used on smartphones, with an estimated 73% share of the smartphone market, as reported in June 2021 [2]. Out of these two, Android is the open-source operating system. This flexibility and openness has proved to be the major reason for the rich contribution of community developers. This has created a healthy ecosystem where Android has flourished in terms of functionality and stability. However, at the same time, this openness has been a major cause for the attraction of developers to build malicious apps, known as mobile malware. These apps threaten the privacy and security of users. As per statistics, in 2019, approximately 350,000 malware were detected every day, and every seven seconds, a new malware was generated. These numbers clearly demonstrate the level of threat and danger faced by smartphone users and businesses. Malware occupies device resources and disturbs their normal functioning. Apart from stealing users’ data and compromising their privacy, it can monitor users’ behavior and learns to cause deeper damage. Thus, it is very important to have effective countermeasures.

At a high level, two techniques [3,4] are used for performing analysis as part of the malware detection process. The first one is a static analysis that performs app code analysis without running it. The focus of this kind of analysis is on understanding the structure of the code along with the functionality [5] it performs. This analysis technique is efficient; however, it can only detect known malware and is unable to provide zero-day protection. In addition, this technique is not able to detect malware that uses obfuscation. Dynamic analysis, on the other hand, observes the behavior of the app when it runs. Being more effective, dynamic analysis can detect unknown malware as well, by learning from observation. The downside of this kind of analysis is that it requires a large amount of resources which, generally, is not feasible for mobile devices. To obtain the benefit of dynamic analysis and overcome this resource requirement issue, malware detection hosted in the cloud as a service [6,7] is proposed. To train the malware detection model, a large amount of data is required from the app providers, while making sure that no sensitive data is shared, in order to protect the user’s privacy. This is one of the greatest challenges.

Permissions have an important role to play in an app’s functioning. In earlier versions of Android, users were forced to allow every permission required by an app at installation time. This created a security concern as, unknowingly, users would allow unnecessary permissions as well. The later versions of Android added dynamic permissions management where the app asks for required permissions at the runtime. The user can then allow or deny the permissions. Since permissions are the first thing any malware will try to exploit, it is proven to be effective to use for malware detection. This study analyzes studies that have been done for malware detection using permissions analysis for Android. Research questions have been defined and answered using a systematic literature review (SLR) protocol. Relevant publications have been searched from various scientific databases. These publications were analyzed through different criteria to include or exclude, along with assessing the quality. Any publication that did not fulfill the selection criteria and quality assessment was discarded, and only high-quality papers were made part of the data extraction and analysis.

The rest of the paper is structured as follows: Section 2 explains the Background. Section 3 shows the Related Work. Section 4 presents the methodology of the research. This section has further sub-sections. Section 4.1 concerns the review protocol. The defined research questions are outlined in Section 4.2. Section 4.3 details the search strategy used to search databases for relevant articles. Exclusion criteria are enumerated in Section 4.5, an assessment of article quality is done in Section 4.6, and the data synthesis process, which is used in the extraction of data and formulating of the result, is explained in Section 4.7, Section 4 is dedicated to discussing the results and Section 5 comprehensively discusses the presented answers to research questions and validity threats. Lastly, the conclusion is presented in Section 6.

## 2. Background

### 2.1. Malware and Android Apps

**Figure 1:**
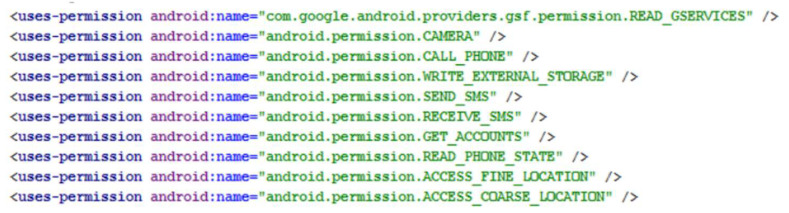
AndroidManifext.xml showing permissions.

By design, every Android app runs within its security boundary. It cannot access data related to other apps or perform sensitive operations by default. The permissions mechanism governs what an app can do. These permissions are declared in the AndroidManifext.xml file for every app. Figure 1 shows a typical AndroidManifest.xml file with declared permissions for the app. In older versions of Android (i.e., earlier than Android 6.0), the user had to accept and allow all permissions required by the app during the installation time. This was a flaw in a way because a user could unknowingly allow a long list of permissions just for the sake of even trying out an app. Android versions 6.0 and greater improved the permissions architecture, where permissions are requested at runtime when required by the app. The user has the ability to either allow or deny.

Since malware is also an app, it needs permission to execute malicious code (e.g., sending SMS at the user’s expense). Since permissions are defined using strings, these strings are used in malware detection. By leveraging the defined permissions, apps are narrowed down and matched with the particular malware sample. Permissions are the first door that needs to be opened for typical malware to get hold of the device. One popular app named T2Expense was removed from the Google Play Store because it was unnecessarily accessing users’ call logs, text messages, and contacts, although it was an expense manager app.

### 2.2. Machine Learning

Machine learning (ML) is a subpart of AI (artificial intelligence), that aims at developing intelligent computer-based systems by using statistical techniques and learning from existing datasets. ML is playing a role in many industries these days for classification and regression tasks. ML models are trained using malware datasets and used in malware detection techniques. ML is at the backend of almost all malware-detecting techniques. Using both static and dynamic analysis, features are extracted from the collected data and datasets are formed. Many datasets are publicly available [8,9] and can be modified and enhanced to be used in building more intelligent and robust ML models.

### 2.3. Types of Malware 

**Figure 2:**
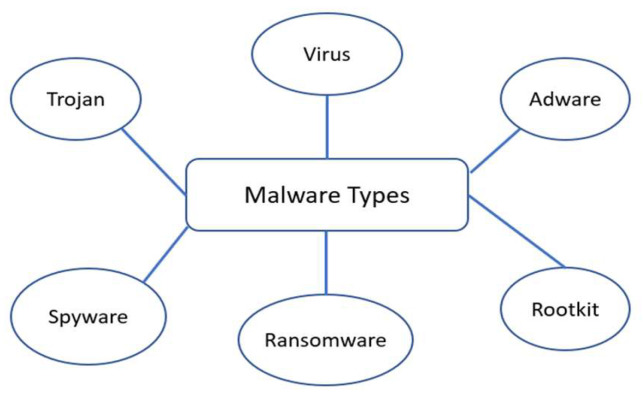
Malware Types.

There are many different types of malware with one common objective: causing damage of all possible kinds to users’ data and assets. Figure 2 shows some of the common types, and the following is a brief description of those: 

Viruses can copy themselves and infect other computers. In addition, they can destroy some files on a computer and can spread themselves to other devices with the help of e-mail programs [10].

Trojans seem to have good functionalities; however, they have hidden functions that can bypass the security layer within the system [10].

Spyware software systems are used to collect information from organizations or people without their knowledge [10].

Ransomware software mostly encrypts the data on a computer with a key, and when the ransom is paid, the key is sent to the user by the hacker so that the user can access his/her data. Unless the ransom is paid, the user cannot access the data on the computer [10].

Rootkit refers to the tools that are used by the attackers after the root-level access is reached. With the help of these tools, the attacker can hide the activities done in the system and maintain root-level access [10].

Adware (a.k.a., advertisement-supported software) creates adverts on the user screen (mostly, on the web browser) and therefore, the developer of the adware is able to obtain profit from these adverts [11].

## 3. Related Work

Our research did not come across an SLR that specifically covers and focuses on malware detection on Android using permission analysis. There are SLRs explaining analysis techniques at a broader level, such as static analysis, hybrid analysis, etc. Pen et al. [12] published an SLR by going through the literature which addresses static analysis techniques for detecting malware for Android OS. The authors divided the static analysis techniques into four categories based on application features. This was done to measure the malware detection capabilities of static analysis. The authors mentioned that using a neural model provides better performance than a non-neural network. In addition, as mentioned by the authors, static analysis is proved to be effective in detecting malware, but some studies report low potential. It has been concluded that using different categories of static analysis together has the potential to achieve better results. Furthermore, there are still challenges faced by static analysis techniques to effectively detect malware on the Android platform. The authors stressed the need to develop novel techniques to alleviate these challenges. As part of future work, the authors intend to promote guidelines to build and promote novel techniques for better malware detection methodologies.

There is a survey paper on permission-based malware detection in Android applications [13]; however, it only focuses on five approaches and the presented work provides very limited knowledge. A similar survey paper exists [14]; however, 13 papers were explained very briefly in this survey study and an in-depth analysis was not performed. Articles that address permission-based malware detection are mostly primary studies and propose new techniques. We could not discover any SLR study that evaluates this particular scope so far. There are other surveys or SLR studies that address the general malware prediction models. Our SLR study is unique because we particularly focus on malware detection using permission analysis.

## 4. Research Methodology

**Figure 3:**
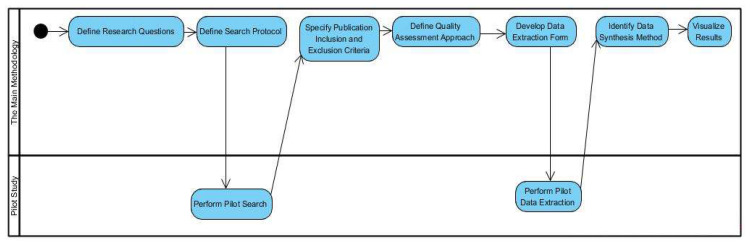
Methodology flowchart.

The following section explains the methodology used in this research. Figure 3 shows the research methodology’s main steps:

### 4.1. Review Protocol

The first and most significant stage before performing research is to define the review protocol. This was accomplished by adopting Kitchenham et al.’s study [15]. The research questions must first be defined. Relevant scientific databases were examined as soon as the research topics were finalized to find relevant research papers. In our study, we searched the following databases:

- ACM
- Google Scholar
- IEEE Xplore
- Science Direct
- Scopus
- Web of Science
- Wiley

Malware detection and prevention is a much-needed area to research and generate solutions. Therefore, there have been many studies done in this area. Some of the databases used returned duplicates of research papers. All non-duplicated studies that were found are included in our review. Later, as per the research questions defined, the data and facts related to the topic were extracted. In the approach, at a high level, the research questions are identified and laid out as part of review planning, followed by protocol construction. The database selection comes next, where we pass the search criteria as input, which is also known as search strings. The next step is to define the selection criteria for primary studies. Finally, the procedure is fine-tuned to ensure that the review protocol is appropriate.

Conducting the review happens in the second phase. This involves selecting the publications from the corresponding databases. Following the selection of articles, data and facts were extracted, yielding information on authors, publication year and kind, and, most importantly, information about the defined research questions. This was followed by a data synthesis procedure, which was then utilized to discuss the articles’ relevance in the context.

The final phase comprised documenting the results and addressing the defined research questions based on the earlier phase. One of the most important parts of this phase is to answer the defined research questions. Wherever necessary, additional studies were searched, selected, and referenced to find the answer to the defined research questions.

### 4.2. Research Questions

The following five research questions are laid out for this study:

- RQ1: What challenges face the permission analysis technique in detecting Android malware?
- RQ2: What possible methods or approaches can be used to mitigate those challenges?
- RQ3: How effective is this approach in the context of new and customized versions of Android?
- RQ4: Out of the studied solutions, which one provides the best result?
- RQ5: Which datasets are used in the primary studies?

### 4.3. Search Strategy

While searching the articles, the focus was to obtain the relevant papers on the topic in which malware detection methodologies and techniques are introduced using permissions analysis. Although there are several techniques introduced, using other aspects of the Android platform, this SLR is focusing on permission analysis. There are several studies published in which authors have used permissions analysis to either introduce a novel technique or have generated improved solutions. “Android malware detection using permissions analysis” was the first keyword used in the search. Since the abstract and conclusion parts of every article provide a good overview, they were used to understand the relevance of the article to our topic. Inclusion and exclusion criteria were applied for filtering the articles. This was followed by using more combinations of search keywords to search more articles, to ensure the inclusion of as many related articles as possible. The following search string combinations were used:

“Android malware detection using permission analysis”;

“permission analysis Android malware”;

“permission analysis” and “Android malware detection”;

“analyzing Android app permission ” and “detecting malware”.

There were 16 related papers found. The categorized count is presented in Table 1. Most of the papers were found and obtained in the IEEE Xplore database.

### 4.4. Prisma Flow Diagram

**Figure 4:**
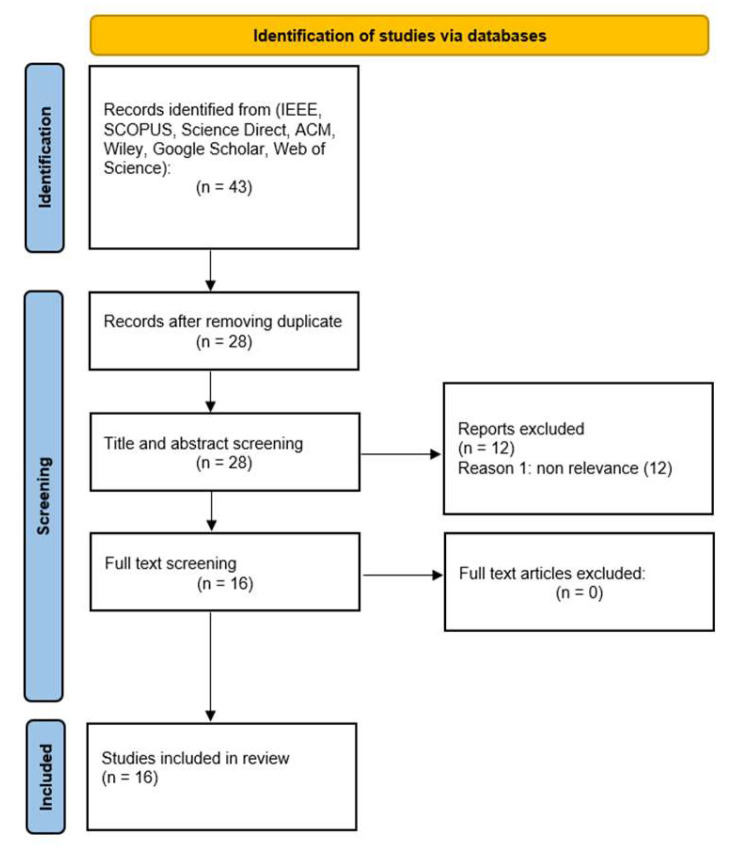
Identification of studies.

In Figure 4, we present how the included articles were selected. For documenting the details, we utilized the PRISMA Flow diagram.

### 4.5. Exclusion Criteria

The identified studies went through the application of exclusion criteria to make sure that irrelevant papers were excluded for this particular SLR. The following details the applied criteria:

- Publications that are not directly related to permission analysis techniques regarding malware detection
- Publications in a language other than English
- Publications that are duplicates
- Publications that have their abstract available only and the full text is not available
- Secondary studies or review papers
- Publication year earlier than 2011

**Figure 5:**
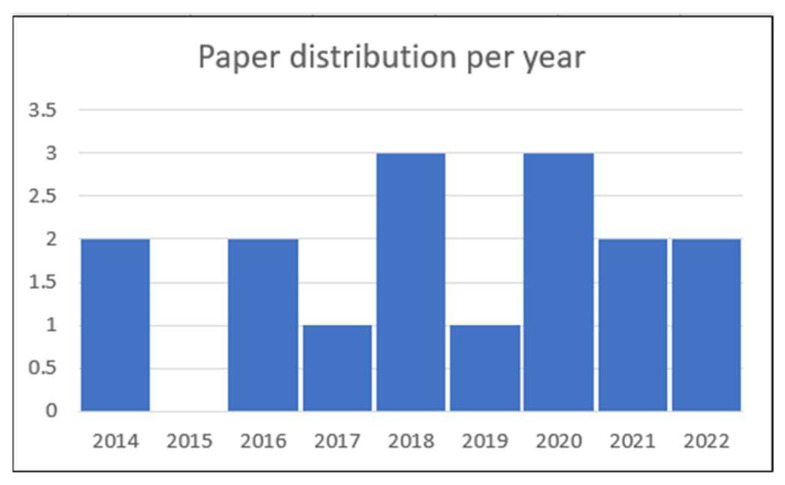
Publications’ distribution per year.

Considering the nature of the topic, many publications were available. This research area is active to improve malware detection accuracy and robustness. Figure 5 shows the distribution of selected papers by year.

The relevant data was taken from articles and synthesized in such a way that the five research questions were effectively addressed. A two-part emphasis was in place when the data was gathered from each article. First, we made sure that the studies we chose met the requirements, and second, that the data we collected could answer research questions. The retrieved and synthesized data were utilized to develop responses to the research questions, and the findings are presented and discussed in the corresponding sections.

### 4.6. Quality Score

After retrieving the publications, they were subjected to a procedure in which the quality of each article was evaluated. This was done to ensure that only high-quality publications were included in this review. The quality evaluation was based on assessment questions derived from a study done by Kitchenham et al. [15].

The quality score was calculated for each publication based on the answer to each question as per the following:

- 1 (if the answer is yes)
- 0 (if the answer is no)
- 0.5 (if the answer is somewhat)

The assessment was comprised of the following questions:

1. Does the study state the aims clearly?
2. Has the scope of the study been clearly defined?
3. Are the variables used in the study reliable?
4. Is the process of research sufficiently covered by the documentation of the study?
5. Has the study effectively answered the questions defined?
6. Does the study result in obstructive findings?
7. Does the study list major outcomes related to reliability and soundness?
8. Does the conclusion coincide with the aims of the study?

**Figure 6:**
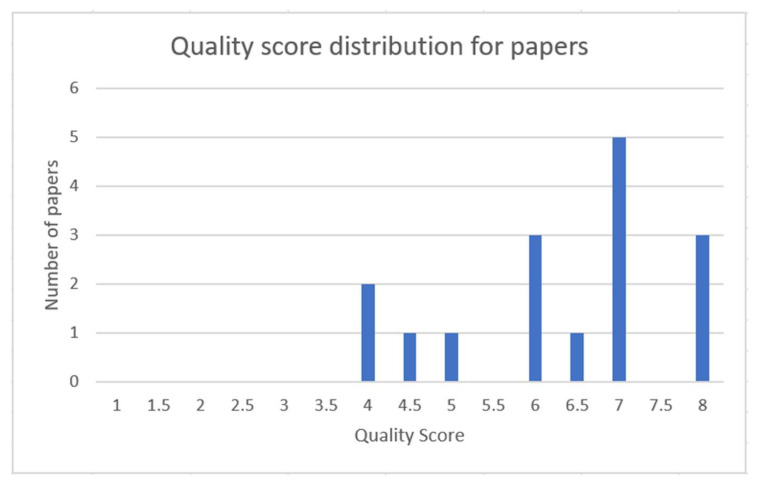
Distribution of the papers quality score basis.

Figure 6 shows the quality score distribution of the papers. The papers that scored 4 or more, which is the threshold value for inclusion in the paper, are part of the study. In this study, all identified papers were included as those are of high quality.

### 4.7. Data Synthesis

To answer the research questions, data synthesis was performed. This was done by aggregating the collected data to extract the required facts. Qualitative data analysis was performed to answer some of the research questions, such as the main challenges faced by malware detection techniques using permission analysis. On the other hand, some questions required us to perform quantitative data synthesis, such as the possible solution to mitigate these challenges. For identifying these solutions, we performed qualitative data synthesis.

## 5. Results

In this section, the response to all five research questions is presented. In its subsection, the response is discussed. Table 2 shows the selected primary papers with extracted data, such as analysis technique used, machine learning classifier, tools, and year of publication.

The following parameters were entered, in line with the data extraction:

- The start-to-end mechanism applied by each study for malware detection
- Analysis technique used
- Machine learning classifier (used or developed if any)
- Additional tools used for the process of analysis or evaluation

Based on the facts and finding, along with analysis, the following section discusses research questions answering:

### 5.1. RQ1—What Challenges Permission Analysis Technique Faces to Detect Android Malware?

Malware infections are on the rise. As per statistics, new malware is produced every few seconds. In addition to this, modern malware development techniques are producing stealthier versions that are difficult to detect. After going through all primary studies, we concluded that there are numerous challenges preventing the development of efficient and effective malware detection methodologies. In [16], the authors argue that combining features to create an optimal feature vector is a challenge, as this would provide higher accuracy. In [17], the authors have described how contrasting pattern length presents a great challenge, because it significantly affects the cost of computing and accuracy. Authors in [21] point out that the limitations of static analysis towards generated fuzzy sets are issues to pay attention to in order to improve the detection process, which can be solved by involving dynamic analysis. In [24], the authors report that correct detection of apps with fewer or no permissions is a challenge as they evade the detection process easily. In addition, the false positive rate (FPR) is an additional issue where several safe apps (social media category) are wrongly classified as malicious apps because of the presence of dangerous permissions pairs. Authors in [26] share a similar challenge, where FPR occurs for apps with fewer permissions, which is added to the challenge of detecting malware that uses obfuscation techniques that make them stealthier. Lastly, in [31], the authors discuss how analyzing dead objects (garbage collection analysis) is another challenge. Malware may leave dead objects as a trace, and this can play a significant role in its correct detection.

Having a consolidated look at the challenges yields the following enumeration of challenges that help in wider understanding:

(a) Preserving user’s privacy during malware detection is a major challenge, no matter what technique is followed. Malware detection with the help of analyzing permissions can be executed in two ways: first, on the device where no information is shared externally, and second, where necessary information is gathered from the device and sent to an external (usually cloud-hosted) service for analysis. Since the device is limited in resources, cloud-based service can be helpful. However, in both cases, there is a privacy concern. In the first case, the malware analysis itself needs elevated privileges so that it can read and decompile .apk files for the sake of permission extraction and further analysis. In the second case, extracting information from the device and sending it to an external service clearly adds more weight to the privacy concern.
(b) Since malware and clean apps might have similar permissions, it is highly likely to end up with FP (false positives) and/or FN (false negatives), where an app is either wrongly classified as malware or wrongly classified as a clean app. This presents yet another challenge to generating an efficient approach to minimizing FP and FN. Malware developers add permissions in the manifest file that are similar to a clean app. This raises the likelihood of fooling the detection process and yielding the wrong result.
(c) Modern malware is developed using state-of-the-art obfuscation technologies, which help them to be even stealthier. This makes it challenging for the detection methodologies to work successfully. In some works in the literature, methodologies are introduced where a hybrid approach is used. In such cases, permissions are evaluated in more depth by analyzing the app behavior as well. However, the malware uses obfuscation to hide various aspects of behavior, such as encrypted external communication. Due to this, dynamic analysis cannot effectively analyze the behavior of the app in the context of declared permissions.

Although, while publishing apps on the Google Play Store, the app goes through several checks before approval, in several instances malicious apps were still able to pass through and were published. Google made it compulsory for app publishers to provide information related to data safety manually [32]. Earlier, this was the permissions section, but it has become the data safety section. The goal was to facilitate the end user’s understanding of permissions, data collection, and usage. Clearly, there is still a high probability that apps can pass through it and be published on the store. In this scenario, the Play Store becomes dependent on what app developers provide as a set of data safety information. According to [33], several developers and app providers were traced lying regarding the permissions their app needed on one of the leading app stores, which is also considered the most secure and sage. This stresses the importance of permissions analysis, even in the presence of these platforms, for malicious detection.

### 5.2. RQ2—What Possible Methods or Approaches Can Be Used to Mitigate Those Challenges?

Considering the diverse nature of devices and the availability of free apps, the need for effective malware detection has grown stronger than ever. As described in RQ1, malware detection meets several challenges. Research is being conducted to mitigate those challenges and produce an effective and robust solution. Out of the mentioned challenges, accuracy is the first concerning one. Accuracy can be affected when FP or FN are faced as a result of the malware detection process. Literature has suggested combining static and dynamic analysis to mitigate this challenge. Since dynamic analysis for malware detection observes the app behavior, it can do a better job when combined with static analysis. However, this leads to a second challenge, which is computing cost. Dynamic analysis requires more computation resources on the device. This could lead to other functioning issues for the device itself. Different studies recommend using dynamic analysis externally by sharing the observed information from the device to an external server. This way, the device would not come under a heavy load of processing. Interestingly, this leads to the third challenge that is discussed in RQ1, which is user privacy. There have been many methodologies and techniques introduced in the literature which detect malware on the Android platform and preserve the user’s privacy at the same time. Although these methodologies use different approaches than permission-based analysis, the objective is common.

Cui et al. [34] proposed PPMDroid, which is a system to detect malware on Android using static and dynamic analyses, while at the same time respecting the privacy of users’ data, phone vendors, and security service providers (SPs). In terms of static analysis, key behavioral footprint features in the app codes, such as remote calls, permission granting requests, constant strings, and package names, are hashed and shared with SPs. SPs compare the received hashes’ values with the malware signatures’ hashes to verify the safety of the applications. Additionally, the similarity of an APK code and resource files are compared with malware samples using hash similarity techniques to detect malicious apps. The usage of one-way hash schemes ensures that phone vendors are not required to needlessly share inner APK code files with SPs to verify their integrity. For dynamic analysis, an encrypted database of malware signatures is created by SPs using secure searchable symmetric encryption (SSE) techniques and stored locally on a user handset. The database is essentially a hash table that stores different malware information, signatures, and behaviors. Next, PPMDroid dynamically analyzes the runtime behaviors of running applications and securely queries the local signatures database to compare the behaviors against malicious ones. This scheme ensures that the application’s code is never shared remotely with security services. Similarly, SPs’ malware signatures are preserved and not exposed to other parties.

Kucuk et al. [35] designed BigBing, which is a cloud-based malware architecture that respects individuals’ privacy while simultaneously employing a scalable big data approach that can scale efficiently with a voluminous number of users’ data. At the core of BigBing is a frontend gateway web interface that users can access to share a list of binary samples with the system. The collection of binary executables is stored in a big data cluster using Hadoop Distributed File System (HDFS). To protect users’ privacy, users are only required to upload partial chunks of their binary samples, which cannot be used to infer sensitive information. Only when distinct chunks of the same binary file are shared by different users can the server reassemble the whole binary file, in which case it is ensured that no user-specific data exists in the file, since it has been assembled from different sources. To share binary files, different users collaboratively contribute different binary chunks by pushing them on a blockchain. At the big data cluster where binary executables are assembled successfully, Apache Cassandra and Apache Spark are used to extract and store relevant features of those binary samples, which are used later to train a classifier for detecting malware.

Hsu et al. [36] proposed a decentralized architecture for training a malware detection model, using federated learning by distributing the task of model training over many collaborating devices while preserving the raw data of each device. Static analysis is performed on a collection of decompiled APK source files as well as related meta-data to extract key information such as requested permissions, API calls, application categories, and descriptions. These key data are encoded as feature vectors to be inputted to a federated learning model based on SVM. To achieve privacy-preserving, federated model training utilizes secure multiparty computation SMPC techniques with additive secrete sharing. The model data is broken into secret shards that are shared securely between the mobile clients. Each client performs local training and exchanges a local model with a server. The server securely computes an aggregated global model, which is sent back to the mobile clients to classify mobile applications based on the existence of malware.

Wei et al. [37] introduced EPMDroid, which is a platform that leverages Intel SGX to guard against Android malware in a privacy-respecting manner. SGX are types of hardware-based trusted execution environments in which an arbitrary program can be executed in isolated and secure environments without compromising its confidentiality, even against privileged access such as OS and BIOS processes. Through SGX, the integrity of a program can be verified while respecting the privacy of the program code and data. SGX suffers from limited memory spaces which limit the amount of data that can be processed at one time without performing extensive I/O operations with a storage component. To address this issue, the authors proposed employing cuckoo filters that can extract a more compact collection of features from the training dataset and store it within CuckooTable, which are hash tables with highly efficient memory spaces. The core of the EPMDroid system consists of applications data providers ADP, mobile devices MD, and malware detection service providers MDSP. ADPs (e.g., app stores) are responsible for submitting new apps to MDSPs, which process and store them in secure SGX enclaves for model training. Similarly, MDs invokes the services of MDSPs to verify the integrity of running apps by conducting inference in secure SGX enclaves.

### 5.3. RQ3—How Effective Is This Approach in the Context of New and Customized Versions of ANDROID?

Android 1.0 was released in 2008 as the first public version for the devices. Since then, many new versions have been released by the vendor. These contain important updates, security patches, and other enhancements. As the devices kept on evolving in terms of capabilities and power, Android kept pace by adding the required enhancements to use that level of power the devices offer [38]. Clearly, using the newly introduced feature over time was bound to new permissions assigned to apps. This implies that the Android future would bring more features and services tied to new permissions. Malware could use those new permissions, along with API calls, to exploit the Android platform in the same way [39]. Equivalently, malware detection methodologies would need to take care of those new permissions by studying their contribution to malicious actions taken by malware. Since permissions are interrelated, newly introduced permissions could lead to some major changes in some of the existing methodologies in the literature.

Since Android is open source [40], it can be customized as required. There are certain brands of Android devices on the market that use a customized version of Android. One example is the case of an Android phone in which certain Android services from the vendor are not available due to some restrictions. These restrictions are imposed by the Android vendor. This forced the phone manufacturer to produce a modified version of Android because it holds a significant smartphone market share. There could be similar cases in the future due to similar or different reasons. However, since the foundation of the Android OS remains the same, there is no change in permissions mechanisms. There is one concern, however: the services that are not allowed by the Android OS on certain phone manufacturers’ devices would force the phone manufacturers to provide an alternative solution (with the same functionality). Those alternative services could be similar in functioning but might require different permission sets that are only required on such specific devices. This could result in the possibility of using a sparse permissions combination, which would make malware detection even more difficult on those devices where the customized version of Android OS is running.

### 5.4. RQ4—Out of the Studied Solutions, Which One Provides the Best Results?

**Figure 7:**
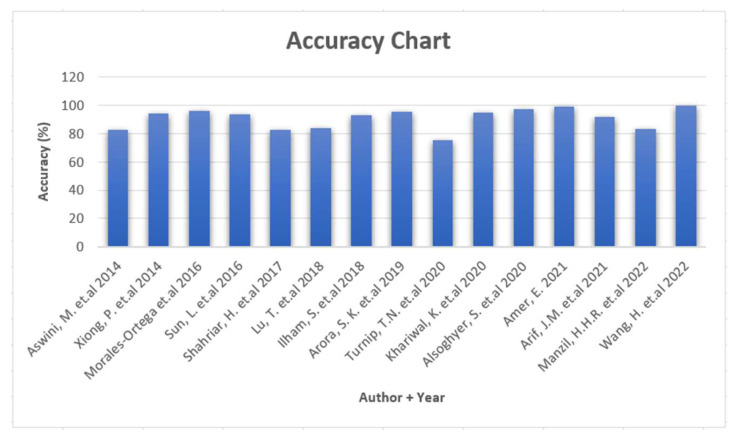
Reported accuracy: ([16,17,18,19,20,21,23,24,25,26,27,28,29,30,31]).

Almost all primary studies which were analyzed for this SLR use accuracy as one common metric to assess the proposed methodology. Several studies showed over 90% accuracy during the evaluation phase. However, at the same time, it was mentioned in the studies that there is a high chance of FP and FN due to various factors. These factors are methodology-specific and discussed in each paper specifically. Some of the studies use other metrics as well, such as F1 Score and AUC (area under curve), but for the sake of a unified answer to this RQ, we would consider accuracy as the assessment metric. Figure 7 shows the accuracy chart for primary studies (except [22], where accuracy was not reported).

As shown in Figure 7, almost all studies were able to achieve over 80% accuracy level. Table 3 shows the malware detection accuracy reported by each study. No accuracy was reported in [22].

As shown in Figure 7 and Table 3 [31], was able to achieve the highest accuracy (99.80). In addition to the highest accuracy, the authors of [31] have also reported the robustness of the proposed solution against techniques that use permissions abuse and obfuscation.

### 5.5. RQ5—Which Datasets Were Used in the Primary Studies?

Table 4 shows the datasets which were used in the primary studies.

## 6. Discussion and Threats to Validity

This section is dedicated to general discussion and explaining possible validity threats.

### 6.1. General Discussion

This SLR systematically reviews the literature on Android malware detection using permission analysis. The main aim was to identify the challenges, proposed solutions, and assessment of the proposed solutions, based on reported metrics. Additionally, some of the mitigation techniques were studied and included in the review along with datasets used in the primary studies. The results were compiled to effectively answer the research questions. Identified challenges could pave the way for further research and improved solutions. Enlisting the datasets used along with machine learning classifier and accuracy would set a clear direction to move into for further research.

Using a hybrid approach (static and dynamic analyses) has proved to be more effective, but computation cost is another major issue. To deal with computation costs, solutions are suggested to gather data from the user’s device and send it to an external service for analysis. This would alleviate the cost burden on the user’s device.

Another challenge in malware detection is preserving users’ privacy during the process. Ensuring that the user’s privacy is intact increases the credibility of the proposed solutions. Increased awareness of users about cyber security has put increased focus on coming up with privacy-ensured solutions. This SLR has also looked at some of the proposed solutions which focus on users’ privacy regarding malware detection.

Another interesting research outlook for malware detection is the use of federated learning (also known as collaborative learning) [42,43] approaches. In traditional machine learning, the datasets are uploaded to a central server, and training is performed using the datasets on this server. However, in federated learning, the algorithm is trained across several edge devices or servers that keep the local samples. With the help of federated learning, data sharing is not required and several stakeholders can work on building a common model. As such, data privacy and data security issues are handled adequately. This type of privacy-preserving federated learning model can protect user data and does not leak user privacy. When the performance is comparable with the centralized models, federated learning-based models must be preferred due to privacy concerns.

Cloud infrastructure as a service (IaaS) can be also vulnerable to malware attacks and, therefore, different techniques using deep learning algorithms, such as convolutional neural networks (CNN), were previously developed for the detection of malware in cloud IaaS [44]. If an Android mobile app uses such a malicious IaaS platform for storage or networking, the mobile app will be also affected. As such, the security of IaaS platforms is also crucial.

These days, ransomware is the most popular, threatening, and malicious type of malware [45]; therefore, more specialized malware prediction models are needed for this type of malware. There are also recent research studies on the use of deep learning-driven image-based malware detection techniques [46,47,48]. These studies help in preserving user privacy and allow researchers to apply state-of-the-art image analysis methods to detect malware signatures. These types of advanced models can improve the performance of the prediction models.

### 6.2. Threats to Validity

There may have been some articles missing due to the lack of synonyms that may have been used in the search. Some observations may have been influenced, because some terminologies were employed differently in different articles. Because all authors were involved in the process and a consensus was formed when analyzing the papers, possible bias in selecting papers was reduced. To lessen the risk of conclusion validity, conclusions were derived by the authors based on discussions and general understanding; as a result, the individual foundation on which results were interpreted was reduced in this study.

## 7. Conclusions

This review investigates the literature about different methodologies used for Android malware detection using permissions analysis. The machine learning classifiers used with reported accuracy were analyzed along with the datasets used. Static and dynamic analyses are used for detecting malware. The studies have shown that static analysis is efficient in terms of performance, but it does not ensure comprehensive detection, as it is not capable of observing the app behavior, whereas dynamic analysis is more effective but is resource-consuming. Some of the studies are using a hybrid approach effectively, achieving the highest accuracy and, at the same time, ensuring that the computation cost remains low.

Obtaining FP and FN during the detection process is another issue that must be further researched. Malware can ask for normal permissions to disguise and fool the detection process and, at the same time, clean apps can be wrongly classified as malware because they ask for sensitive permissions. Proposed solutions have attempted to tackle this using different approaches, and there have been good results. However, as time passes, more malware is appearing with new disguising techniques that present a major challenge.

As part of future work, the outcomes of this SLR would be enhanced by focusing on the role of deep learning in malware detection on Android, along with privacy-preserving methodologies for users.

## Figures and Tables

**Table 1:** Count of papers returned by databases.

Database	Papers Count
IEEE Xplore	12
ACM	2
Scopus	2

**Table 2:** Selected primary study papers.

Ref.	Title	Analysis Tech. Used	Machine Learning Classifier(s) Used/Developed	Tools Used (If Any)	Year
[16]	Droid permission miner: Mining prominent permissions for Android malware analysis	Static Analysis	Naïve Bayes, AdaBoost, Random forest	Androguard (to generate human-readable manifest file)	2014
[17]	Android malware detection with contrasting permission patterns	Hybrid permission profile (normal, malware, common)	Enclamald	Weka (for classifier comparison)	2014
[18]	Native malware detection in smartphones with android OS using static analysis, feature selection and ensemble classifiers	Static Analysis	SMO used by SVM, Random Forest, Random Committee with Random Tree, and Random Committee with Random Forest	Android AssetPackaging Tool (AAPT) for obtaining features	2016
[19]	SigPID: significant permission identification for android malware detection	Static Analysis	SVM (Support Vector Machine)	N/A	2016
[20]	Android malware detection using permission analysis	Static Analysis	N/A	Apktool for decompiling .apk build files	2017
[21]	A Two-Layered Malware Detection Model Based on Permission for Android	Static Analysis	Random Forest	Eclipse (for java development)	2018
[22]	API and permission-based classification system for Android malware analysis	Static Analysis	N/A (to be used in future work)	YARA (for identifying malware using pattern matching)	2018
[23]	Permission Based Malware Detection in Android Devices	Static Analysis	Random Forest, SVM	Androguard (for extracting permissions)	2018
[24]	PermPair: Android Malware Detection Using Permission Pairs	Static Analysis	N/A	N/A	2019
[25]	Android Malware Classification Based on Permission Categories Using Extreme Gradient Boosting	Static Analysis	XGBoost	Androguard (for extracting permissions from .apk files)	2020
[26]	IPDroid: Android Malware Detection using Intents and Permissions	Static Analysis	Random Forest, SVM, Naive Bayes	VirusTotal (to test malicious app dataset), Apktool (for permission extraction)	2020
[27]	On the Effectiveness of Application Permissions for Android Ransomware Detection	Hybrid (Static and Dynamic analysis)	Random Forest (RF), Decision Trees, Sequential minimal optimization algorithm (SMO), Naive Bayes (NB)	Apktool (to decompile .apk file and get manifest information)	2020
[28]	Permission-Based Approach for Android Malware Analysis Through Ensemble-Based Voting Model	Static Analysis	Random Forest, MLP, AdaBoost, SVM, Decision Tree	N/A	2021
[29]	A static analysis approach for Android permission-based malware detection systems	Static Analysis	Random Forest, kNN, MLP, J48, Adaboost (Random Forest has the highest accuracy)	WEKA (machine learning tool for evaluation)	2021
[30]	COVID-Themed Android Malware Analysis and Detection Framework Based on Permissions	Static Analysis	Decision Tree, Random Forest	Androguard (for decompiling .apk files), APKAnalyzer (for permission extraction)	2022
[31]	You are what the permissions told me! Android malware detection based on hybrid tactics	Hybrid (Static and Dynamic analysis)	TextCNN	AHAT (for heap analysis)	2022

**Table 3:** Accuracy that was reported by each study.

Ref.	Title	Accuracy(%)
[16]	Droid permission miner: Mining prominent permissions for Android malware analysis	82.48
[17]	Android malware detection with contrasting permission patterns	94.38
[18]	Native malware detection in smartphones with android OS using static analysis, feature selection and ensemble classifiers	96.26
[19]	SigPID: significant permission identification for android malware detection	93.62
[20]	Android malware detection using permission analysis	82.33
[21]	A Two-Layered Malware Detection Model Based on Permission for Android	83.60
[22]	API and permission-based classification system for Android malware analysis	NA (not reported)
[23]	Permission Based Malware Detection in Android Devices	93
[24]	PermPair: Android Malware Detection Using Permission Pairs	95.44
[25]	Android Malware Classification Based on Permission Categories Using Extreme Gradient Boosting	75.55
[26]	IPDroid: Android Malware Detection using Intents and Permissions	94.73
[27]	On the Effectiveness of Application Permissions for Android Ransomware Detection	96.90
[28]	Permission-Based Approach for Android Malware Analysis Through Ensemble-Based Voting Model	99.3
[29]	A static analysis approach for Android permission-based malware detection systems	91.60
[30]	COVID-Themed Android Malware Analysis and Detection Framework Based on Permissions	83
[31]	You are what the permissions told me! Android malware detection based on hybrid tactics	99.80

**Table 4:** Datasets used in primary studies.

Ref.	Dataset(s) Used
[16]	Contagiodump
[17]	Custom dataset was developed using apps downloaded from SlideME and Pandaapp. In addition, a dataset published by Zhou et al. [41] was used
[18]	Derbin
[19]	Dataset was custom developed after downloading 5494 randomly selected apps from Google Play. The article does not state the availability or other details about this dataset
[20]	Malgenome project and Contagio
[21]	Datasets collected from Baidu application market and North Carolina State University’s Android Malware Genemo Project
[22]	N/A (future works would include using machine learning)
[23]	AMD Projects
[24]	Genome, Derbin, Koodous, Contagio, PwnZen
[25]	Koodous
[26]	Genome, Derbin, Koodous
[27]	Dataset-R (from HelDroid, RansomProper, Virus Total, and Koodoud)Dataset-B (from Google Play)
[28]	Derbin, MalGenome
[29]	Drebin, Androzoo
[30]	The authors collected COVID-related apps from different sources including Google Play and GitHub, and the dataset was custom-created using feature elimination techniques. The dataset has not been released for public use.
[31]	As reported by the authors, the dataset was custom-made after collecting and processing more than 12,364 malware apps and 9344 clean apps from Google Play. The dataset has not been released for public use.

## Data Availability

Not applicable.
